# Phytohormone Cross-Talk Synthesizes Glycoalkaloids in Potato (*Solanum tuberosum* L.) in Response to Aphid (*Myzus persicae* Sulzer) Infestation under Drought Stress

**DOI:** 10.3390/insects11110724

**Published:** 2020-10-23

**Authors:** Peter Quandahor, Yuping Gou, Chunyan Lin, Mohammed Mujitaba Dawuda, Jeffrey A. Coulter, Changzhong Liu

**Affiliations:** 1 College of Plant Protection, Gansu Agricultural University, Lanzhou, No. 1 Yingmen Village, Anning District, Lanzhou 730070, China; quandooh@yahoo.com (P.Q.); gouyp1988@163.com (Y.G.); linchunyan0905@163.com (C.L.); 2 Department of Horticulture, Faculty of Agriculture, University for Development Studies, P.O Box TL 1882 Tamale, Ghana; mmdawuda@yahoo.com; 3 Department of Agronomy and Plant Genetics, University of Minnesota, St. Paul, MN 55108, USA; jeffcoulter@umn.edu

**Keywords:** potato, phytohormones, α-chaconine, α-solanine, hydrogen peroxide

## Abstract

**Simple Summary:**

Potato (*Solanum tuberosum* L.) is a vegetable crop that plays a major role in global food security. However, its production and sustainability are adversely affected due to aphid infestation. The green peach aphid, *Myzus persicae* (Sulzer), poses a significant threat to potato plants globally due to its high production of honeydew and transmission of viruses. Other researchers reported that drought severity could result in an outbreak of insect pests such as aphids. Accordingly, understanding the mechanism of host plant defense against aphids under drought stress is a major concern for insect pest management. This study was conducted to examine the cross-talk of phytohormones in potato glycoalkaloids’ defense against green peach aphids under greenhouse conditions. The results showed that under drought conditions, the cross-talks of phytohormones do not only function as signal hormones, but also modify host plant secondary metabolites to defend against sap-sucking insects. Many potato cultivars may activate phytohormones under drought stress; however, only host plants with a greater level of secondary metabolites may be able to defend against aphid attack. This research will provide a scientific basis for the development of potato varieties with high yield, improved quality, and drought and pest resistance.

**Abstract:**

Potato production is adversely affected by aphid infestation across the globe. Understanding the mechanism of host plant defense against aphids under drought stress is paramount for insect pest management. This study was conducted to examine the cross-talk of phytohormones in potato glycoalkaloids’ defense against green peach aphids under greenhouse conditions. A 3 × 2 × 2 factorial experiment comprising three potato cultivars (Qingshu 9, Longshu 3, and Atlantic) and two levels each of water availability and aphid infestation was conducted. The results show that under drought stress, green peach aphids thrive well on host plants, which contain a relatively high water content. The resistant cultivar DXY, which exhibited a higher level of phytohormones, also demonstrated higher α-chaconine and α-solanine contents in both leaf and root, under drought and aphid stress. Conversely, the susceptible cultivar QS9, which exhibited a lower level of phytohormones, also demonstrated low α-chaconine and α-solanine contents in both leaf and root, under drought and aphid stress. The DXY cultivar, which possessed high resistant traits such as α-chaconine and α-solanine, can be used in areas where green peach aphid infestation is a major setback.

## 1. Introduction

Drought stress is a major challenge in agriculture that improves the performance of herbivorous insects by modifying the nutrition and palatability of host plants [1]. The reduction in the stomatal aperture under drought stress improves host plants’ natural resistance to aphids’ performance, by inducing secondary metabolites [2,3]. Plants of the Solanaceae family produce variations of secondary metabolites comprising glycoalkaloids, which negatively influence the reproductive potential and population growth of aphids [4]. The main glycoalkaloids that occur in commercially cultivated potato cultivars are α-chaconine and α-solanine [5]. The concentrations of glycoalkaloids differ greatly in the various parts of potato [5]. Preferably, high glycoalkaloids levels in the leaves of potato plants act as natural defense against sup-sacking insects, whereas a low concentration in tubers also decreases health risks of consumers [5]. It is speculated that the modification of host plant secondary metabolites against aphid attack may be due to phytohormone biosynthesis [6].

Plants under stress conditions are controlled by the synthesis of phytohormones, comprising jasmonic acid (JA), salicylic acid (SA), and abscisic acid (ABA), which can cross-talk to induce plant natural defenses against aphids’ performance [7]. The accumulation of ABA caused abscission and a reduction in the stomatal aperture, which increase water use efficiency, as well as the modification of some secondary metabolites [8,9,10]. Research on the effect of *Bemisia tabaci* (silverleaf whitefly) on Arabidopsis indicated that SA acid inhibited the population growth and reproduction potential of this insect. Phytohormones such as JA and its precursor *cis* (+) 12-oxophytodienoic acid (OPDA) are also reported to act as plant defense mechanisms against insect pests [11].

Potato (*Solanum tuberosum* L.) is a vegetable crop that plays a major role in global food security [12]. However, its production and sustainability globally are adversely affected due to aphid attack [13,14]. The responses of aphids to drought stress have been widely reported [15,16] and still lack consensus. Koricheva et al. [17] reported that aphid population increased more on drought-stressed cultivars than on well-watered cultivars, whereas Huberty and Denno [18] reported conflicting results under similar conditions. Particularly, only few field trials support the notion that the aphid population increases on drought-stressed cultivars; an experimentally imposed water deficit, nonetheless, often negatively influences aphid population abundance [18]. The green peach aphid, *Myzus persicae* (Sulzer), is considered a major pest of potato plants due to its high production of honeydew and transmission of viruses [19,20]. Other researchers reported that drought severity could result in an outbreak of insect pests such as aphids [21,22]. Thus, understanding the mechanism of host plant defense against aphids under drought stress is paramount for insect pest management. This study was based on the hypothesis that the changes in phytohormones such as JA, SA, ABA, and OPDA will synthesize potato glycoalkaloids to inhibit aphid reproductive performance and population growth under drought stress. This study was conducted to examine the cross-talk of phytohormones in potato glycoalkaloids’ defense against green peach aphids under greenhouse conditions. This research will provide a scientific basis for the development of potato varieties with high yield, improved quality, and drought and pest resistance.

## 2. Materials and Methods

### 2.1. Growth Conditions and Plant Materials

Growth conditions and plant materials were used as described in our previous study [20]. The experiment was conducted in a greenhouse (day temperature 25–35 °C, night temperature 18–22 °C, daytime RH 45–55%, light intensity 15,000–18,000 lux) at the College of Plant Protection, Gansu Agricultural University, Lanzhou, China, during the summer of 2019. The daily temperature of 35 °C was the highest recorded during the experiment, but this was encountered only once. Higher daily temperatures recorded over the period of the experiment were 33, 35, 32, and 30 °C, which occurred on the 31st, 46th, 57th, and 83rd day, respectively, throughout the experiment. However, the average daily temperature recorded for the period of the experiment was 26.5 °C. Mini tubers of three different potato cultivars were obtained from the Gansu Seed Research Laboratory, China, for the experiment. The potato genotypes were Qingshu 9, Longshu 3, and Atlantic, and were characterized as tolerant, moderately tolerant, and sensitive, respectively, to drought stress. The pots (20 cm diameter, 9.5 cm deep) used for the experiment were filled with 2 kg of loamy soils. Every pot was provided with one mini tuber and basic agronomic practices such as watering and weed control were conducted periodically. Uniform water was supplied to every experimental pot until the commencement of the drought treatment (40 d after planting).

### 2.2. Aphid Culture

The aphids (*Myzus persicae*) used for this experiment were collected from a potato cultivation area of Gansu Agricultural University. The collected adult aphids were cultured on potato plants in the laboratory (LD 16:8 h photocycle at 19 ± 1 °C). The aphids were raised until six months, when the experiment began. Nymph aphids of the same age class (about 9th generation age class) were collected for the experiment.

### 2.3. Experimental Design and Treatments

The experimental design was described in our previous study [20]. A 3 × 2 × 2 factorial experiment in a split-split plot design with three replications was conducted in a greenhouse. The treatments comprised three potato cultivars (Qingshu 9, Longshu 3, and Atlantic) and two levels each of water availability and aphid infestation. The three potato cultivars were allocated to the main plots. The main plots were split into subplots for the drought stress and well-watered treatments. The split plots were further split for the allocation of the aphid and no aphid treatments. Six pots per experimental unit were assigned to a water treatment. A total of 216 pots were used for the experiment. In each experimental unit, six plants were sampled for data collection, giving a total of six subsamples in each of the three replications for each treatment. The drought-free plants were plants growing in a moisture content of 100% of soil capacity, whereas the drought-stressed plants were plants growing in a moisture content of 30% of soil capacity. The well-watered treatments were watered effectively to keep the soil water content at 100% soil capacity. For the drought-stressed treatments, soil water content was allowed to drop gradually (by withholding watering) for 20 d, and then kept at 30% of soil capacity until the end of the experiment. The water levels throughout the experiment (92 d) per treatment were maintained by weighing the pots every 5 d, and adding water capacity. A Delta-T Theta Probe ML2 (Delta-T Devices, Cambridge, UK) was used to measure soil water content daily to maintain consistency among pots. This process continued until the experiment was over.

### 2.4. Determination of Aphid Performance

Six potato seedlings of each treatment were infested with 20 nymph aphids of the same generation (about 9th generation age class). The number of nymphs per plant was assessed by counting the number of nymphs produced until the 20th day after infestation. The number of nymphs produced on a plant was divided by the number of adults per plant, at 20 d after infestation, to assess the number of nymphs per adult.

### 2.5. Determination of Hydrogen Peroxide (H_2_O_2_), Malondialdehyde (MDA), and Protein Contents in Aphids

The aphids from each treatment, which weighed 0.1 g, were collected and immediately frozen in liquid nitrogen and three replicates were taken for each treatment. The collected aphid samples were homogenized in 0.05 M phosphate buffer solution with pH 7.8, 0.1 mM ethylenediamine tetraacetic acid, and 1% polyvinylpyrrolidone. The centrifugation of the crude homogenates was performed at 10,000× *g* for 15 min at 4 °C. The supernatant was used to measure the absorbance of H_2_O_2_ and MDA at 510 nm against reagent blank and 532 nm, respectively, by the spectrophotometry method (Shimadzu UV-2450, Arlington, MA, USA). The H_2_O_2_ and MDA contents were determined following the methods of Zhu et al. [23]. Protein content of the aphid supernatants was determined using the method described by Bradford [24].

### 2.6. Leaf Abscission, Plant Stomatal Conductance, and Water Use Efficiency

Leaf abscission was determined by counting the number of leaves (from six plants) of each cultivar at 60 d after planting. Stomatal conductance was also determined by measuring the fifth to tenth terminal mature leaves (from six plants) from the bottom of the potato seedling at 30 d after infestation. Stomatal conductance of plants was measured with a Li-Cor 6400 gas exchange system (6400-40; Li-Cor Inc., Lincoln, NE, USA) between 07:30 and 10:30 h. Water-use efficiency was measured as described by Des Marais et al. [25].

### 2.7. Determination of Potato Aboveground Biomass

Potato aboveground biomass was determined as described previously by Bañón et al. [26]. Aboveground fresh shoot weight was determined immediately after harvest. The harvested fresh shoots were dried at 80 °C for 72 h to express the aboveground dry weight per plant.

### 2.8. Determination of Hormones in Leaf and Root Samples

The contents of JA, SA, ABA, and OPDA were determined as previously described by Stingl et al. [27]. In brief, fresh leaf and root samples of each cultivar were collected from six plants in each experimental unit. The samples were wrapped with aluminum foil and immediately frozen in liquid nitrogen. Subsequently, the samples were stored in a freezer at −80 °C. Tissue samples weighed at 0.5 g were prepared for each sample. The tissues were homogenized with liquid nitrogen and each sample was added with a 1.5 mL aliquot of extraction buffer (2:1:0.005, isopropanol/water/concentrated HCl). Samples were agitated for 30 min at 4 °C. An amount of 1.5 mL of CH_2_Cl_2_ was added, followed by agitation for another 30 min and then centrifugation at 13,000× *g* for 5 min. After centrifugation, two phases were formed and plant debris was in the middle of two layers. The aqueous phase was discarded and the lower layer was collected and concentrated in a rotary evaporator, and re-solubilized in 200 μL of 60% MeOH. JA, SA, ABA, and OPDA concentrations were quantified by comparison of sample peak areas with standard curves of reference phytohormones (Agilent Chemical Co., Santa Clara, CA, USA).

### 2.9. Determination of Glycoalkaloid Content in Potato

Glycoalkaloids (α-chaconine and α-solanine) contents were determined as described by Dao and Friedman [5]. Potato leaf and root samples were collected from six plants in each experimental unit. Subsequently, 20 mL of 5% aqueous acetic acid was added to 50 g sample. A Sorvall mixer was then used to homogenize the samples at high speed for 2 min. The suspension was poured into a flask and a magnetic stirrer was used to stir the suspension for 3 h. The sample was filtered under vacuum by using Whatman No. 4 filter paper. The extraction of the filtrate was repeated for another 3 h with an additional 15 mL of 5% acetic acid. The filtrates were poured into a separatory funnel and the pH was set at 10–11 with ammonium hydroxide. The alkaline extract was separated twice with 20 mL of water-saturated butanol. An air vacuum rotovaper was used to dry evaporate the butanol extracts. Colorimetric analysis was conducted for the glycoalkaloids after the filtrate was re-dissolved in 5 mL of absolute methanol. Absorption was recorded at the wavelength of 600 nm (solution color changes to blue and then gets lighter). The amount of potato total glycoalkaloids was calculated based on the α-solanine standard curve. The results of the analyses are given as mg per g of fresh matter.

### 2.10. Statistical Analysis

Three-way ANOVA was used to compare uninfested and infested plants for ABA, JA, OPDA, SA, α-chaconine, and α-solanine contents, leaf water use efficiency, leaf abscission, aboveground fresh, stomatal conductance, and aboveground dry weight. Two-way ANOVA was used to analyze the number of nymphs per plant, number of nymphs per adult, and aphid MDA and H_2_O_2_ contents. All data were checked for normality and homogeneity of variances. The data collected were analyzed by the analysis of variance using SPSS statistics software (Version19.0 for Windows, SPSS, Chicago, IL, USA). Treatment means were separated using Fisher’s least significant difference test (*p* < 0.05). The results are represented as means ± standard error.

## 3. Results

### 3.1. Aphid Performance

**Figure 1:**
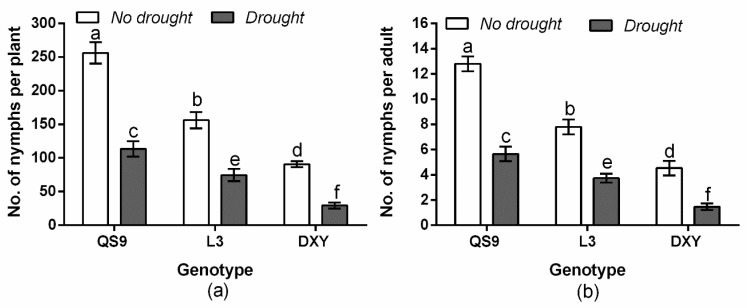
Number of nymphs per plant (**a**) and number of nymphs per adult (**b**) in three potato cultivars grown under well-watered or drought conditions with and without peach aphid infestation. Data represent the mean ± standard error of three replicates. Lowercase letters indicate treatment means that are statistically significantly different according to the least significant difference test (*p* < 0.05).

The results of this experiment showed a significant (*p* < 0.01) variety × drought interaction effect on the number of nymphs per plant and the number of nymphs per adult (Figure 1). Generally, the number of nymphs per plant and the number of nymphs per adult were higher on well-watered plants than on drought-stressed plants. The number of nymphs per plant on well-watered plants and drought-stressed plants was highest on QS9 and lowest on DXY. The number of nymphs per adult on well-watered plants (59.8%) and on drought-stressed plants (28.9%) was also highest on QS9 and lowest on DXY (22.9 and 7.3%, respectively). Thus, among the genotypes, the greatest number of aphids per plant and aphids per adult occurred on QS9, whereas DXY had the lowest under drought or no drought conditions.

### 3.2. Effect of Drought Stress on Aphid H_2_O_2_ and MDA Contents

**Figure 2:**
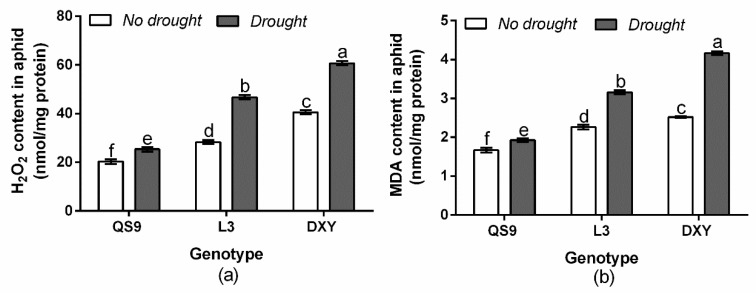
Changes in hydrogen peroxide (H_2_O_2_) content (**a**) and malondialdehyde (MDA) content (**b**) in green peach aphid reared on three potato cultivars grown under well-watered or drought conditions with and without peach aphid infestation. Data represent the mean ± standard error of three replicates. Lowercase letters indicate treatment means that are statistically significantly different according to the least significant difference test (*p* < 0.05).

To associate the functional attributes of H_2_O_2_ and MDA with drought and aphid tolerance of the cultivars, the MDA and H_2_O_2_ contents were analyzed. Accordingly, the contents of H_2_O_2_ and MDA significantly increased compared with the control under drought stress (Figure 2). The content of H_2_O_2_ in aphids reared on QS9, L3, and DXY increased by 23.4, 48.6, and 55.2%, respectively, under drought stress. Drought stress also increased the MDA content in aphids reared on QS9, L3, and DXY by 13.3, 25.9, and 51.1%, respectively.

### 3.3. Genotypic Variation of Morphological and Physiological Response to Drought Stress and Aphid Treatments

**Figure 3:**
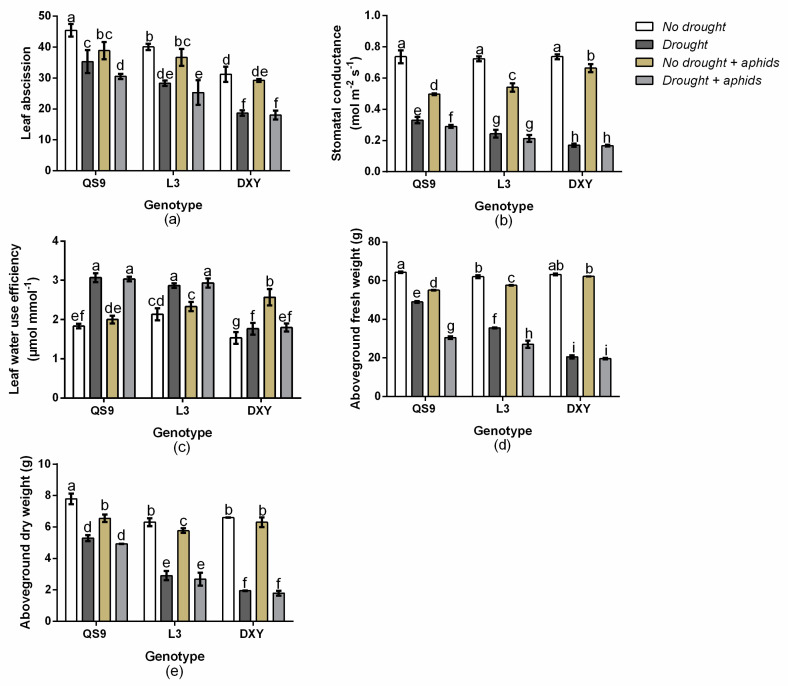
Changes in leave abscission (**a**), stomatal conductance (**b**), leaf water use efficiency (**c**), aboveground fresh weight (**d**), and aboveground dry weight (**e**) in three potato cultivars grown under well-watered or drought conditions with and without peach aphid infestation. Data represent the mean ± standard error of three replicates. Lower case letters indicate statistically significantly differences between cultivars within the same water treatment and aphid treatment by the least significant difference test (*p* < 0.05).

There was a significant (*p* < 0.03) genotype × drought × aphid interaction effect on leaf water use efficiency, leaf abscission, and aboveground fresh weight. However, stomatal conductance and aboveground dry weight were not affected (*p* > 0.05). Under drought stress, leaf abscission of QS9, L3, and DXY decreased by 22.2, 29.3, and 40.2%, respectively, and by 14.4, 8.5, and 6.4% under aphid stress (Figure 3a). Drought stress also decreased stomatal conductance of QS9, L3, and DXY by 55.2, 66.3, and 76.9%, respectively, and by 32.5, 25.3, and 9.9% under aphid stress (Figure 3b). Leaf water use efficiency of QS9, L3, and DXY increased by 40.2, 25.5, and 13.2%, respectively, and by 8.3, 8.5, and 30.2% under aphid stress (Figure 3c). Drought stress also decreased aboveground fresh weight of QS9, L3, and DXY by 23.7, 42.7, and 68.9%, respectively, and by 14.3, 7.1, and 1.6% under aphid stress (Figure 3d). Aboveground dry weight of QS9, L3, and DXY increased by 15.0, 53.8, and 70.6%, respectively, and by 15.9, 8.3, and 4.3% under aphid stress (Figure 3e). The lowest decrease in leaf abscission, stomatal conductance, aboveground fresh, and dry weight occurred in QS9, under drought stress. Conversely, DXY had the lowest decrease in leaf abscission and stomatal conductance, and aboveground fresh and dry weight under aphid stress. Moreover, the highest and lowest water use efficiency also occurred in QS9 and DXY, respectively, under drought stress.

### 3.4. Genotypic Variation of Pytohormone Defense Induced by Drought Stress and Aphid Treatments

**Figure 4:**
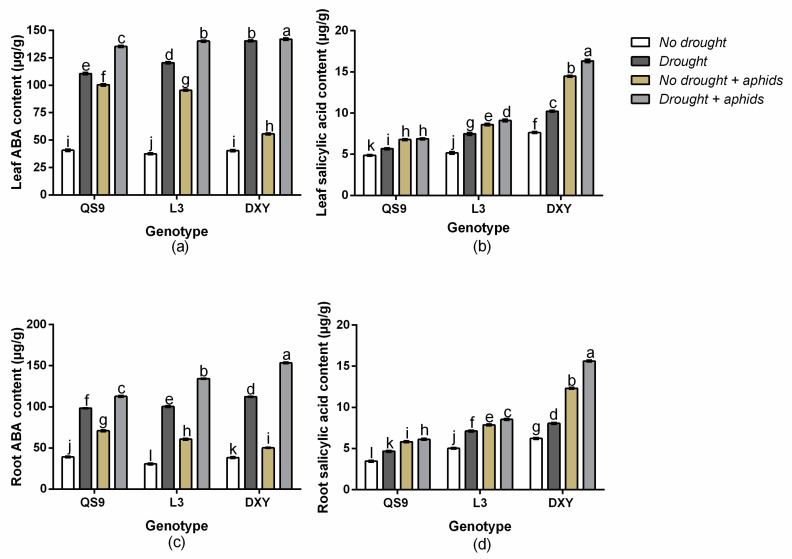
Changes in leaf abscisic acid (ABA) content (**a**), leaf salicylic acid (**b**), root ABA content (**c**), and root salicylic acid (**d**) in three potato cultivars grown under well-watered or drought conditions with and without peach aphid infestation. Data represent the mean ± standard error of three replicates. Lower case letters indicate statistically significant differences between cultivars within the same water treatment and aphid treatment by the least significant difference test (*p* < 0.05).

There was a significant (*p* < 0.01) genotype × drought × aphid interaction effect on leaf ABA content, leaf SA content, root ABA content, and root SA content. Under drought stress, the leaf ABA content of QS9, L3, and DXY increased by 63.1, 68.9, and 71.2%, respectively, and by 60.7, 59.3, and 27.4% under aphid stress (Figure 4a). Drought stress also increased the leaf SA content of QS9, L3, and DXY by 14.1, 25.4, and 30.8%, respectively, and by 28.0, 39.9, and 47.2% under aphid stress (Figure 4b). The root ABA content of QS9, L3, and DXY increased by 60.1, 65.8, and 69.4%, respectively, under drought stress and by 44.7, 49.5, and 23.7% under aphid stress (Figure 4c). The root SA content of QS9, L3, and DXY also increased by 22.7, 25.7, and 29.4%, respectively, under drought stress and by 36.0, 40.5, and 49.4% under aphid stress (Figure 4d). The greatest leaf and root ABA content occurred on DXY plants, under drought stress. Conversely, the lowest ABA content in both leaf and root occurred in DXY under aphid stress. Moreover, DXY had the greatest SA content in both leaf and root under drought and aphid stress.

**Figure 5:**
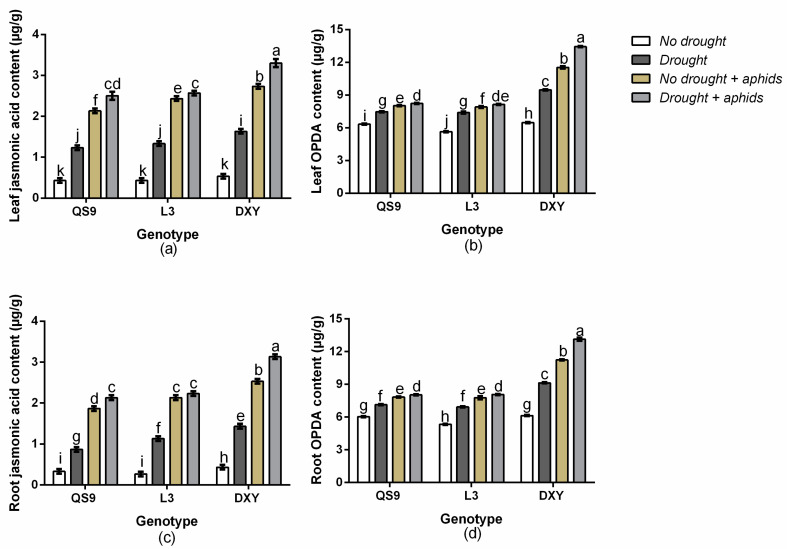
Changes in leaf jasmonic acid content (**a**), leaf *cis*(+) -12-oxophytodienoic acid OPDA content (**b**), root jasmonic acid content (**c**), and root OPDA content (**d**) in three potato cultivars grown under well-watered or drought conditions with and without peach aphid infestation. Data represent the mean ± standard error of three replicates. Lower case letters indicate statistically significant differences between cultivars within the same water treatment and aphid treatment by the least significant difference test (*p* < 0.05).

There was a significant (*p* < 0.01) genotype × drought × aphid interaction effect on leaf OPDA content, root JA content, and root OPDA content. However, leaf JA content was not affected (*p* > 0.05). Under drought stress, the leaf JA content of QS9, L3, and DXY increased by 64.8, 67.3, and 67.5%, respectively, and by 79.6, 80.4, and 82.1% under aphid stress (Figure 5a). Drought stress also increased the leaf OPDA content of QS9, L3, and DXY by 15.1, 23.8, and 31.6%, respectively, and by 21.1, 28.6, and 43.9% under aphid stress (Figure 5b). The root JA content of QS9, L3, and DXY increased by 61.5, 69.7, and 76.4%, respectively, under drought stress and by 82.1, 82.8, and 87.5% under aphid stress (Figure 5c). The root OPDA content of QS9, L3, and DXY also increased by 15.4, 23.0, and 32.8%, respectively, under drought stress and by 22.9, 31.3, and 45.4% under aphid stress (Figure 5d). The greatest JA and OPDA content in both leaf and root occurred in DXY plants, under drought and aphid stress. Conversely, the lowest JA and OPDA contents in both leaf and root occurred in QS9, under drought and aphid stress.

### 3.5. Genotypic Variation of Glycoalkaloid Defense Induced by Drought Stress and Aphid Treatments

**Figure 6:**
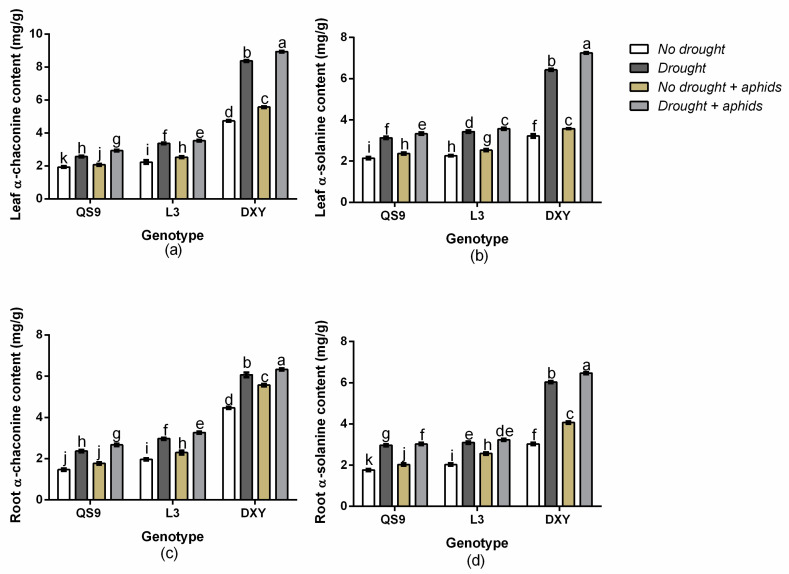
Changes in leaf α-chaconine content (**a**), leaf α-solanine content (**b**), root α-chaconine content (**c**), and root α-solanine content (**d**) in three potato cultivars grown under well-watered or drought conditions with and without peach aphid infestation. Data represent the mean ± standard error of three replicates. Lower case letters indicate statistically significant differences between cultivars within the same water treatment and aphid treatment by the least significant difference test (*p* < 0.05).

There was a significant (*p* < 0.01) genotype × drought × aphid interaction effect on leaf α-chaconine, leaf α-solanine, root α-chaconine, and root α-chaconine. Generally, both α-chaconine and α-solanine contents were higher in leaf than in root. Under drought stress, the leaf α-chaconine content of QS9, L3, and DXY increased by 24.6, 33.6, and 43.6%, respectively, and by 6.4, 11.8, and 17.4% under aphid stress (Figure 6a). Drought stress also increased the leaf α-solanine content of QS9, L3, and DXY by 31.1, 31.5, and 49.8%, respectively, and by 6.7, 9.4, and 12.1% under aphid stress (Figure 6b). The root α-chaconine content of QS9, L3, and DXY increased by 26.3, 30.1, and 39.3%, respectively, under drought stress and by 14.4, 16.9, and 19.7% under aphid stress (Figure 6c). The root α-solanine content of QS9, L3, and DXY also increased by 33.6, 39.7, and 40.4%, respectively, under drought stress and by 13.1, 20.7, and 25.5% under aphid stress (Figure 6d). The greatest α-chaconine and α-solanine content in both leaf and root occurred in DXY plants, under drought and aphid stress, whereas the lowest α-chaconine and α-solanine content in both leaf and root occurred QS9 under drought and aphid stress.

## 4. Discussion

The responses of aphids to drought have been extensively studied [15,19], though there are still uncertainties. To clarify these uncertainties, researchers have presented the plant stress hypothesis, which affirms that drought stress increases the population of insect pests [28]. In the present study, aphids generally performed better on the drought-free plants, compared with the drought-stressed plants, across all cultivars. It appears that sup-sacking insects such as aphids thrive well on host plants, which contain a relatively high water content. Although drought stress negatively affected aphid performance across genotypes, comparatively, the green peach aphids performed better on QS9 plants, under drought stress, compared with the other cultivars. This is because drought stress possibly activated ABA in the QS9 plants, which induced abscission and stomatal closure to improve water use efficiency to maintain a high turgor pressure that helped the aphids to efficiently feed on the phloem. Moreover, the greatest aboveground biomass also occurred on QS9 plants, under drought stress. The DXY plants, which exhibited low water use efficiency and aboveground biomass under drought stress, demonstrated high resistance to the green peach aphid, compared with the other cultivars. Other studies showed that the performance of aphids under drought stress may vary depending on the type of plant, resistance mechanism, level of stress, and type of insect species [22,29]. Noticeably, the lowest abscission and stomatal closure occurred in QS9 plants; yet, their water use efficiency was the highest across all cultivars, under drought stress. Abscisic acid is reported to play a major role in increasing water use in plant metabolism by improving hydraulic conductivity in plants subjected to drought [30]. This suggests that the activation of ABA probably improved the hydraulic conductivity in plants, which transported enough available soil water to improve water use efficiency.

Variations in host plant physical and chemical composition can have important consequences on herbivore population dynamics [31]. It is reported that potato varieties differ in the volatile profiles in their headspace and these differences elicit different behaviors from the green peach aphid [32]. Drought stress can modify the chemical composition of host plants, which can influence aphid population abundance in diverse ways [32,33]. Moreover, plants are known to contain secondary metabolites that are capable of affecting aphid performance [34]. We speculate that plants’ natural defense against aphid attack and water availability contributes significantly to the outcome of aphid performance. The drought-sensitive genotype (DXY) exhibited higher resistance to aphids under both water conditions. This indicates that the drought-sensitive cultivar may contain secondary metabolites that act as repellants to the peach aphid or is less preferred by the aphids due to its high loss of water. Though drought stress negatively influenced aphid performance across cultivars, the tolerant cultivar (QS9) was the most susceptible host because the aphids survived better on it. This is an indication that potato resistance to water deficit might influence their resistance to aphid attack. The DXY cultivar can be utilized to protect against losses in potato production in regions where peach aphids are major pests. Previous studies reported that the evolutionary advantage of a rapid generation time of an aphid is that they can rapidly increase population growth under favorable environmental conditions [35]. Their reproductive performance is correlated to several environmental factors [35]. Photoperiod and temperature were the first environmental factors to be discovered as important factors which contribute to aphids’ reproductive performance [36]. However, the present study speculates that plants’ natural defense against aphid attack and water availability contributes significantly to the outcome of aphid performance. The temperature, light intensity, and relative humidity in the greenhouse probably had some impact on the present results, as reported by previous studies [35,36]. As reported by Asin [37], temperature above 30 °C reduced the adult reproductive capacity of aphids. However, a lower mortality rate and higher reproductive performance were observed at an average temperature of 27.5 °C. This is an indication that temperature above 30 °C probably influenced the mortality rate and reproductive performance of the green peach aphids in the current experiment.

Plants under stress conditions are controlled by the synthesis of phytohormones, comprising JA, SA, and ABA, which can cross-talk to induce plant natural defense against aphids [7]. Several research works have shown that accumulation of ABA can cause the modification of some secondary metabolites in host plants under drought stress [9,10]. The present study showed that JA, SA, ABA, and OPDA in both root and leaves were synthesized under drought and aphid stress, compared with their control plants, across all cultivars. Comparatively, DXY plants, which showed resistance to green peach aphid, had the greatest JA, SA, ABA, and OPDA contents in both root and leaves under drought stress. Previous studies reported that the cross-talk of JA, SA, ABA, and OPDA contributes to plant defense against herbivore performance under stress [9]. Moreover, ABA plays a major role in plant resistance to insect pest attack by subduing SA-dependent defenses to induce JA-dependent defenses, which consequently inhibit the elevation of the production of some secondary metabolites [9,10]. Here, we speculate that the increase in ABA, JA, SA, and OPDA contents in the resistant cultivar, DXY, did not suppress the glycoalkaloids but cross-talked to synthesize them against green peach aphid, under drought stress. The reduction in the stomatal aperture under drought stress improves host plant resistance to aphid performance by inducing secondary metabolites [2,3].

Relative to all water treatments, α-chaconine and α-solanine contents were higher in drought-stressed plants compared with well-watered plants. Noticeably, the cross-talk of ABA, JA, SA, and OPDA modified the glycoalkaloids in all cultivars against the green peach aphid performance, under drought stress. The greater resistance to the green peach aphid, compared to their corresponding control plants, demonstrated this modification. It seems that the activation of α-chaconine and α-solanine contents in all the potato cultivars under drought stress played a positive role in their resistance to the green peach aphid, as synthesized by the phytohormones’ cross-talk. However, the cultivar which demonstrated a greater level of α-chaconine and α-solanine contents showed the greatest resistance to the green peach aphids. This shows that the variation of total α-chaconine and α-solanine contents in potato cultivars can contribute greatly to their resistance to aphid attack, under drought stress. It is reported that host plants induce glycoalkaloids when under environmental or drought stress [38]. For example, the content of glycoalkaloids in commercial potato was increased under a prolonged day length [39]. The upper leaves of potato plants have been reported to possess a greater content of glycoalkaloids, compared with the lower leaves [40]. However, brown leaves of tomato plants were described to possess a greater content of glycoalkaloids compared with fresh leaves [41]. The resistant cultivar DXY, which exhibited higher phytohormones, also demonstrated greater α-chaconine and α-solanine contents in both leaf and root, under drought and aphid stress. Conversely, the susceptible cultivar QS9, which exhibited lower phytohormones, also demonstrated low α-chaconine and α-solanine contents in both leaf and root, under drought and aphid stress. This suggests that the resistance trait exhibited by the DXY plants was possibly due to the synthesis of α-chaconine and α-solanine contents by the phytohormone signaling pathways. The role of secondary metabolites in *Brassicaceae* interaction with aphids is controlled by the phytohormone signaling pathways [6].

Variations in host plant chemical composition can have a negative impact on herbivore attack [31]. The physiology of plants can be altered due to water stresses [42], which can positively [33] or negatively [43] influence the performance of aphids. It is apparent that the extreme water loss of the drought-sensitive cultivar DXY may have contributed significantly in its resistance to the green peach aphid, by decreasing xylem absorption. However, this cultivar greatly inhibited the performance of the aphids under the well-watered condition, confirmed by high H_2_O_2_ and MDA contents accumulated in the aphids reared on it. This is an indication that the greater modification of α-chaconine and α-solanine contents in the DXY plants possibly inhibited aphid phloem absorption. Preferably, cultivated potato plants contain a greater content of glycoalkaloids in leaves to serve as a natural defense against herbivore attack and a lower content in tubers to avoid a potential health hazard to consumers [5]. In this study, the α-chaconine and α-solanine contents were higher in leaves than in roots across all cultivars. Thus, the DXY cultivar can provide natural defense against aphid attack without endangering the health of consumers.

## 5. Conclusions

The results of this study show that under drought stress, green peach aphids thrive well on host plants, which contain a relatively high water content. The QS9 plants, which exhibited greater water use efficiency, also demonstrated poor resistance to green peach aphid, compared with the other cultivars. Under drought conditions, the cross-talks of phytohormones such as JA, SA, ABA, and OPDA do not only function as signal hormones, but also modify host plant secondary metabolites to defend against sap-sucking insects. Drought stress triggered the accumulation of ABA, which induced abscission and stomatal closure to improve water use efficiency to maintain a high turgor pressure that helped the aphids to efficiently feed on the phloem. ABA also played a major role in the resistance cultivar’s response to the green peach aphid attack by subduing SA-dependent defenses to induce JA-dependent and its precursor’s, OPDA, defenses, which consequently elevated the production of the glycoalkaloids in the potato plants. Many potato cultivars may activate phytohormones under drought stress; however, only host plants with a greater level of secondary metabolites may be able to defend against aphid attack. The greater α-chaconine and α-solanine contents in the DXY plants inhibited aphid performance under both water treatments. Most importantly, the DXY cultivar, which demonstrated high resistance to the green peach aphid, can be used in areas where the green peach aphid is a major challenge to potato cultivation.
